# Water Extract of *Potentilla discolor* Bunge Improves Hepatic Glucose Homeostasis by Regulating Gluconeogenesis and Glycogen Synthesis in High-Fat Diet and Streptozotocin-Induced Type 2 Diabetic Mice

**DOI:** 10.3389/fnut.2020.00161

**Published:** 2020-09-15

**Authors:** Tiange Li, Rui Chang, Huijuan Zhang, Min Du, Xueying Mao

**Affiliations:** ^1^Beijing Advanced Innovation Center for Food Nutrition and Human Health, Key Laboratory of Precision Nutrition and Food Quality, Key Laboratory of Functional Dairy, Ministry of Education, College of Food Science and Nutritional Engineering, China Agricultural University, Beijing, China; ^2^Department of Animal Sciences, Washington State University, Pullman, WA, United States

**Keywords:** *Potentilla discolor* Bunge, gluconeogenesis, glycogen synthesis, insulin sensitivity, type 2 diabetes

## Abstract

*Potentilla discolor* Bunge, as a traditional Chinese medicine, exhibits many phytochemical activities. The aim of the present study was to investigate the effects of *Potentilla discolor* Bunge water extract (PDBW) and its underlying mechanisms on gluconeogenesis and glycogen synthesis in high-fat diet/streptozotocin (HFD/STZ)-induced type 2 diabetic mice. LC-MS/MS analyses of PDBW identified 6 major compounds including apigenin-7-O-β-D-glucoside, epicatechin, quercetin 3-O-β-D-glucuronide, kaempferol-3-O-β-D-glucopyranoside, scutellarin, and quercitrin. In the study, a mouse model of type 2 diabetes was induced by 4-week HFD combined with STZ (40 mg/kg body weight) for 5 days. After oral administration of PDBW at 400 mg/kg body weight daily for 8 weeks, the mice with type 2 diabetes showed significant decrease in the levels of fasting blood glucose and glycated hemoglobin A1c (HbA1c), and increase in the insulin level. PDBW improved the glucose tolerance, insulin sensitivity and lipid profiles. Furthermore, PDBW inhibited the mRNA levels of key gluconeogenic enzymes [phosphoenolpyruvate carboxykinase (PEPCK) and glucose-6-phosphatase (G6Pase)] in liver. PDBW also promoted glycogen synthesis by raising the liver glycogen content, decreasing the phosphorylation of glycogen synthase (GS) and increasing the phosphorylation of glycogen synthase kinase3β (GSK3β). Besides, PDBW induced the activation of protein kinase B (Akt) and AMP-activated protein kinase (AMPK), which might explain changes in the phosphorylation of above enzymes. In summary, PDBW supplementation ameliorates metabolic disorders in a HFD/STZ diabetic mouse model, suggesting the potential application of PDBW in prevention and amelioration of type 2 diabetes.

## Introduction

Type 2 diabetes (T2D) is one of the largest global health problems affecting over 400 million people worldwide in recent years (1). As a complex and progressive metabolic disease, T2D is characterized by chronic hyperglycemia resulting from defects in insulin secretion and action due to β-cell dysfunction and insulin resistance in target organs (2). T2D requires long-term glycemic control and can trigger a steep increase in the risk of severe complications including diabetic nephropathy, cardiovascular and stroke (3). In clinical practice, metformin and thiazolidinediones were often used to treat diabetes while they have side effects such as digestive discomfort, increased cardiovascular morbidity and potential toxicities (4). Therefore, it is of great urgency to find effective methods on the blood glucose control to improve T2D.

Insulin sensitivity of peripheral tissues is critical for preventing hyperglycemia after meals (5). Liver plays an important role in the maintenance of glucose homeostasis by regulating glucose storage *via* glycogen synthesis (glycogenesis) and glucose production *via* the breakdown of glycogen (gluconeogenesis) (6). In diabetic individuals, glycogenesis is reduced while gluconeogenesis is strongly elevated, which leads to the increased rate of hepatic glucose output and causes hyperglycemia ultimately (7). The reduced glycogenesis is associated with the phosphorylation of glycogen synthase kinase-3 (GSK3), which subsequently inactivates glycogen synthase (GS) (8). Gluconeogenesis is mainly modulated by phosphoenolpyruvate carboxykinase (PEPCK) and glucose-6-phosphatase (G6Pase). PEPCK and G6Pase are up-regulated during the development of T2D (9). Glucose utilization in liver is mainly regulated by phosphoinositide-3-kinase and its activity is regulated by protein kinase B (PI3K/Akt). PI3K/Akt signaling pathway decreased hepatic glucose output mainly through inducing the phosphorylation of GSK3, thereby stimulating glycogen synthesis (10). AMPK is a key regulator of energy balance (11). AMPK activation can lower blood glucose level and inhibit lipid accumulation by decreasing gluconeogenesis and fatty acid synthesis, and increasing fatty acid oxidation in liver (12). Therefore, maintaining the homeostasis of hepatic glycogenesis and gluconeogenesis is important for T2D prevention and treatment.

The traditional Chinese medicine, *Potentilla discolor* Bunge (PDB), has a long history of clinical application for the treatment of hepatitis, diarrhea or traumatic hemorrhage (13). In recent years, several benefits of PDB and its extracts, such as anti-tumor, anti-cancer, and anti-ulcerogenic activity, have been reported (14). Especially, PDB has drawn much attention in protection against T2D. The extract of PDB dose-dependently reduced the blood glucose levels in alloxan-induced diabetic mice (15). Four weeks' treatment with water extract of PDB ameliorated the development of hyperglycemia and hyperlipidemia in obese mice (16). However, the underlying mechanisms on the hypoglycemic effects of PDB remain largely undefined.

The purpose of this study was to investigate the effects of *Potentilla discolor* Bunge water extract (PDBW) on hepatic glucose homeostasis in high-fat diet and streptozotocin (HFD/STZ)-induced type 2 diabetic mice. To study the hypoglycemic mechanisms of PDBW, the expression of key factors regulating glycogenesis and gluconeogenesis and the alteration of PI3K/Akt and AMPK signaling were evaluated.

## Materials and Methods

### Materials

The air-dried plant of PDB was provided by Tangxian Dandelion Tea Manufacturing Co., Ltd (Tang country, Hebei Province, China) and was identified and authenticated by the taxonomist of Beijing University of Chinese Medicine. Streptozotocin (STZ) and insulin were purchased from Sigma-Aldrich (St. Louis, MO, USA). The mouse insulin enzyme-linked immunosorbent assay (ELISA) kit was purchased from Mercodia (Uppsala, Sweden). RIPA buffer, phosphatase cocktails and protease for western blot, and BCA protein assay kit were purchased from Beyotime Biotech (Haimen, Jiangsu, China). Primary antibodies against p-GS (Ser641), GS, p-GSK3β (Ser9), GSK3β, p-Akt (Ser473), Akt, p-AMPK (Thr172), and AMPK were purchased from Cell Signaling Technology (Beverly, MA). The primary antibody against β-actin was purchased from Biosynthesis Biotechnology (Beijing, China). The anti-rabbit secondary antibody was purchased from Beyotime Biotech (Haimen, Jiangsu, China). All other chemicals were of analytical grade and obtained from Sinopharm Chemical Reagent Co., Ltd (Shanghai, China).

### Preparation of PDBW

One kilogram of PDB was soaked in 10 L of boiling water for 1 h, and the process was repeated twice. The combined extract was filtered using double gauze and then centrifuged before concentrated on a rotary evaporator under reduced pressure at 40°C. The concentrate was lyophilized to obtain the powder.

### LC-MS/MS Analysis of Components From PDBW

Components in PDBW were analyzed with a LC-MS/MS system. Chromatographic separation was performed using an Agilent 1,290 Infinity II UPLC system (Agilent, Santa Clara, CA, USA) equipped with an Agilent Eclipse XDB-C18 column (100 mm × 2.1 mm i.d., 3.5 μm). The column temperature was set at 35°C and the UV absorption wavelengths were set as 254 and 320 nm, respectively. Elution were accomplished on a gradient of formic acid (0.1%) in water (mobile phase A) vs. formic acid (0.1%) in acetonitrile (mobile phase B) at a flow rate of 0.3 mL/min and the injection volume was 10 μL. An optimal gradient elution program was applied to separate the components effectively: 0–15 min, 5–90% B; 15–20 min, 90% B. MS/MS analysis was operated using a high resolution mass spectrometer (Q-Exactive Focus, Thermo Fisher Scientific). The MS data were acquired from an electrospray ionization (ESI) source in positive and negative ion mode, respectively. The parameters of the source were set as follows: nebulizer gas pressure 45.00 psi; electrospray voltage 4,000 V; fragmentor 150 V; desolvation gas (nitrogen > 99.99%) flow 600 L/h; desolvation temperature 350°C and source temperature 100°C; target mass m/z 400; scan range m/z 100–1,500. Data acquisition processing was carried out using Thermo Fisher Xcalibur workstation (Xcalibur software, version 4.0).

### Animal Treatments

The C57BL/6J mice (5 weeks old, male) were purchased from Beijing Vital River Laboratory Animal Technology Co., Ltd. (Beijing, China). Additionally, mice were housed at a controlled temperature (22 ± 1°C) and humidity (40 ± 10%) with a 12-h light-dark cycle. All mice were weighed and given free access to food and water. Body weight and food consumption were monitored throughout the study. All the experimental procedures, animal care and handling were performed according to the guidelines provided by the Animal Care Committee and approved by the Ethics Committee of China Agricultural University (Approval No. KY160018).

After 1-week adaptation, the diabetic mice were established with some modification of methods described previously (17). Mice were fed by a normal chow diet (CON group, KeAoXieLi Feed Co., Ltd., Beijing, China) (*n* = 10) or a high-fat diet (KeAoXieLi Feed Co., Ltd., Beijing, China) (*n* = 20) for 4 weeks. The normal chow diet contains 19.38% (w/w) protein, 45.79% (w/w) carbohydrate, and 4.48% (w/w) fat, whereas the high-fat diet contains 26.2% (w/w) protein, 26.30% (w/w) carbohydrate, and 34.90% (w/w) fat. After fasting overnight, high-fat-fed mice were injected with streptozotocin (40 mg/kg body weight, dissolved in freshly prepared 100 mmol/L citrate buffer) for consecutive 5 days and the mice in the CON group were injected with an equal amount of citrate buffer. The mice with fasting blood glucose level more than 11.1 mmol/L were considered diabetic and then selected for study. Then the diabetic mice were divided into two groups and fed with high-fat diet (T2D group, *n* = 8) or high-fat diet with PDBW at 400 mg/kg body weight (T2DP group, *n* = 8) by intragastric administration for another 2 months. The CON and T2D group were orally administered with an equal amount of sterile physiological saline. At the end of the experiment, all mice were euthanized after overnight fasting. Blood samples were collected and stored at 4°C overnight. Afterwards, samples were centrifuged (4°C, 1,300 × g, 15 min) to obtain serum for analysis. The livers were collected, rinsed and stored at −80°C.

### Measurement of Fasting Blood Glucose and Serum Insulin Levels

After overnight fasting, the blood glucose levels from tail vein of mice were measured using a glucose meter (Roche Diagnostics, Mannheim, Germany). After treatment with PDBW for 8 weeks, the serum insulin level was determined by mouse insulin enzyme-linked immunosorbent assay (ELISA) kit (R&D Systems, Minneapolis, MN, USA).

### Measurement of Glycated Hemoglobin, Serum Lipid Parameters, and Hepatic Glycogen Content

The level of glycated hemoglobin (HbA1c) was determined using glycated hemoglobin kit (Enzyme-linked Biotechnology, Shanghai, China) according to the manufacturer's instructions. The serum lipid parameters [triglyceride (TG), total cholesterol (TC), high-density lipoprotein (HDL-C), low-density lipoprotein (LDL-C) cholesterol, and free fatty acids (FFA)] were measured using commercially diagnostic kits (Jiancheng Bioengineering Institute, Nanjing, China). Glycogen contents in the liver were determined using a colorimetric assay kit (Jiancheng Bioengineering Institute, Nanjing, China).

### Oral Glucose Tolerance Test (OGTT), Intraperitoneal Insulin Tolerance Test (IPITT), and Pyruvate Tolerance Test (PTT)

OGTT was performed after treatment with PDBW for 7 weeks. Mice were orally given glucose at a dose of 2.0 g/kg body weight after an overnight fast, and blood glucose levels were measured at 0, 30, 60, 90, and 120 min after administration using a glucose meter. IPITT and PTT were performed after treatment with PDBW for 8 weeks. After an overnight fast, mice were injected intraperitoneally with insulin at 1.0 U/kg body weight or sodium pyruvate solution at 2.0 g/kg body weight, respectively. Then blood glucose levels were measured at 0, 30, 60, 90, and 120 min. The results of OGTT, IPITT, and PTT were expressed as area under the curve (AUC) calculated according to the previous study (18).

### Real-Time Quantitative Polymerase Chain Reaction

Total RNA was extracted from liver using Trizol reagent (Tiangen Biotech, Beijing, China). First strand cDNA was synthesized from 10 μL of total RNA in a 20 μL reaction volume using 5 × All-In-One RT Master Mix kit (Abm, Richmond, BC, Canada) according to the manufacturer's instructions in a C1000 Thermal Cycler (Bio-Rad, Hercules, USA). The SYBR green-based RT-PCR assay was implemented with a Techne Quantica real-time PCR system (Hangzhou Bioer Technology, Hangzhou, China). The RT-PCR program was as follows: 40 cycles of 95°C for 180 s, 95°C for 30 s, 60°C for 30 s and 72°C for 30 s. The specific primers which include both sense and antisense were showed in Table S1. The mRNA expression was normalized to the house keeping gene GAPDH. Data was presented as the fold change relative to the control group.

### Histological Analysis

The liver was fixed in 4% paraformaldehyde solution and embedded in paraffin wax followed by sectioning into 4 μm thickness. Sections were then stained with hematoxylin-eosin and examined with an electron microscope (Olympus Corporation, Tokyo, Japan).

### Western Blot Analysis

The liver was lysed in RIPA buffer with phosphatase cocktails and protease. The homogenates were then centrifuged at 12,000 × g at 4°C for 15 min and then the supernatants were collected. After measurement of protein concentration by quantification using BCA protein assay reagent (Beyotime Biotech, Haimen, Jiangsu, China), equal amounts of protein samples were separated by 10% sodium dodecyl sulfatepolyacrylamide gel electrophoresis and transferred to polyvinylidene fluoride membranes on a wet transfer apparatus (Bio-Rad, Hercules, USA). The membranes were blocked by 5% non-fat milk power in tris-buffered saline containing 0.1% Tween-20 (TBS-T) for 2 h at room temperature, followed by incubation with primary antibodies overnight at 4°C. After washed by TBS-T, the membranes were incubated with a peroxidase conjugated secondary antibody for 2 h at room temperature. Protein bands were detected by an enhanced chemiluminescenece method using enhanced chemiluminescence (ECL) reagents (Millipore, Billerica, MA, USA). The band intensities were analyzed using software ImageJ 1.47v (Wayne Rasband, Bethesda, MD, USA). All protein expressions were normalized by β-actin.

### Statistics Analysis

Data were expressed as means ± standard error of the mean (SEM) and analyzed by SPSS 20.0 software. Significant differences (*p* < 0.05) between means were evaluated with one way ANOVA followed by Duncan's multiple-comparison test.

## Results

### Identification of Components in PDBW

**Figure 1:**
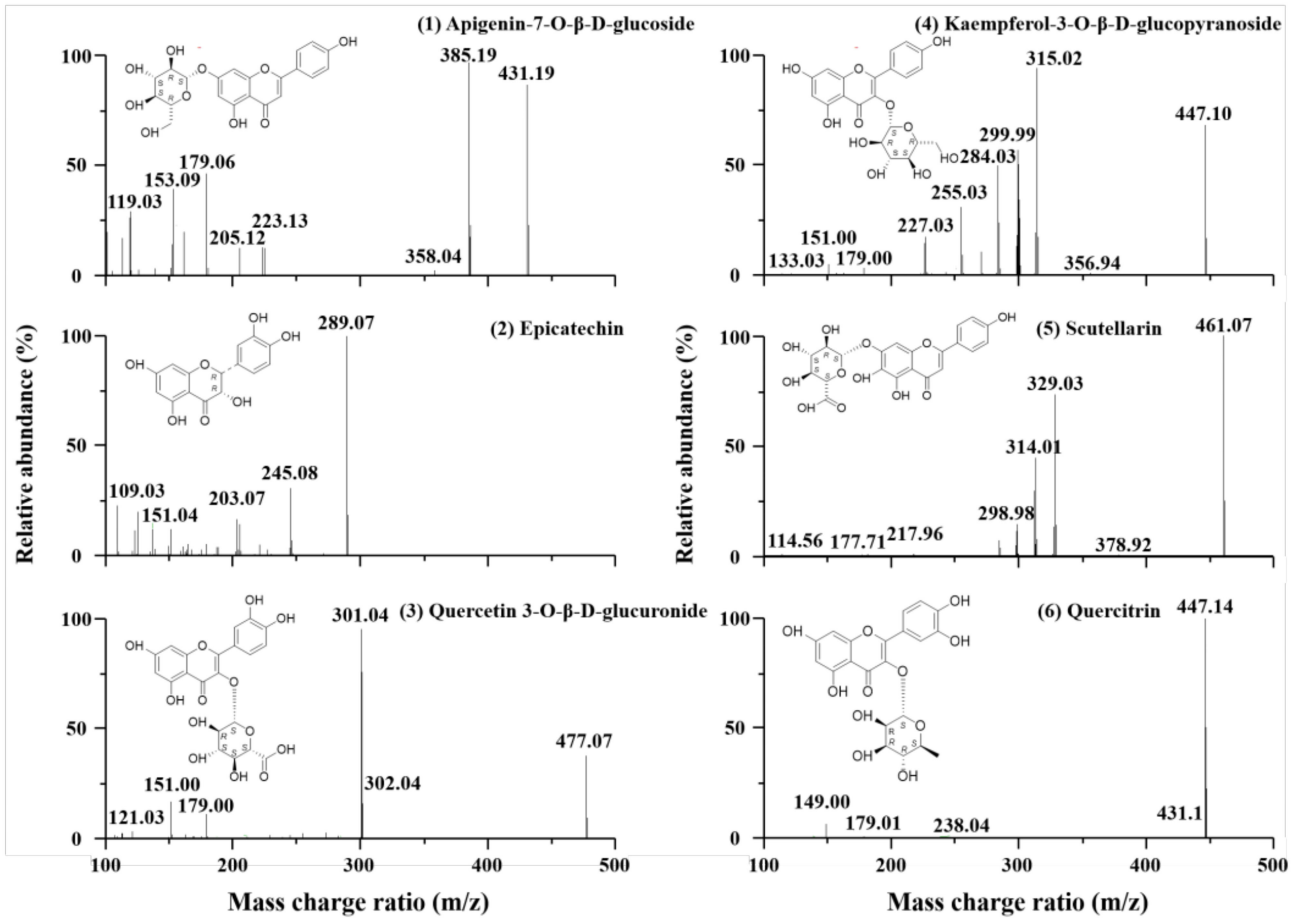
LC-MS/MS spectra of main components in *Potentilla discolor* Bunge water extract (PDBW): (1) apigenin-7-O-β-D-glucoside, (2) epicatechin, (3) quercetin 3-O-β-D-glucuronide, (4) kaempferol-3-O-β-D-glucopyranoside, (5) scutellarin, and (6) quercitrin.

To identify the main components of PDBW, LC-MS/MS was applied to characterize their chemical structures. As shown in Figure S1A, UV chromatograms of the components in PBDW at 254 and 320 nm suggested that six major components can be well-separated within 20 min and their λ_max_ were all at 254 nm. The characteristic ions of the detected components presented in MS total ion chromatogram profiles in negative ion mode were stronger than those in positive ion mode (Figure S1B). The characteristic fragment ions of compounds 1–6 are shown in MS/MS spectra (Figure 1). For fragmentation analysis, taking compound 4 as an example, the quasi-molecular ([M–H]^−^) ion at m/z 447.10 was identified as C_21_H_20_O_11_ by element matching. By comparing the UV absorption, it was speculated as a glycoside compound. As shown in Figure 1, the characteristic ion of aglycone radical [Y_0_-H]^−^ at m/z 284.03 by loss of 163 Da (C_6_H_11_O_5_, a glucose) from the precursor ion [M–H]^−^ had higher abundance than that of aglycone [${\text{Y}}_{0}^{-}$] at m/z 285.03 (not marked in Figure 1), which suggested that the aglycone and glucose were linked by 3-O glucosidic band. Prominent ions at m/z 315.02 [(M–H)–^1, 5^X_0_]^−^ and m/z 299.99 [(M–^0, 1^X_0_)^−^] was obtained from the quasi-molecular ([M–H]^−^) ion and molecular ([M^−^]) ion by fracturing of chemical bond at the 1,5-position and 0,1-position of the glucose, respectively. Besides, the characteristic fragment ions of the aglycone radical [Y_0_-H]^−^ at m/z 284.03 was also matched to that of a flavonol (Mass Spectrum data from the SciFinder Database: scifinder.cas.org). For flavonols, there were some low abundance ions that were generated from the fragmentation pathways of retro-Diels–Alder (RDA) cleavage from the 1,4-position of their C-ring. The fragment ions at m/z 151.00 [^1, 3^A^−^], m/z 133.03 [^1, 3^B^+^] and m/z 179.00 [^1, 2^A^−^] indicated that there were two hydroxyl substituents on ring A, one on ring B and one on ring C. In addition, flavonols are more likely to lose basic radicals or some small molecular fragments, like H (1), ${\text{CH}}_{3}^{\cdot }$ (15), H_2_O (18), CO (28), H+CO (29), CO+${\text{CH}}_{3}^{\cdot }$ (43), and CO+H_2_O (46), H+2CO (57), *etc*., in their structures and yield the basic fragment peaks. The diagnostic fragment ions of compounds 4 at m/z 255.03 [(Y_0_-H)–(H+CO)]^−^ and m/z 227.03 [(Y_0_-H)–(H+2CO)]^−^ were derived from the aglycone radical. Based on the retention time and UV absorption, MS/MS spectrum library and other literature data, this component was identified as kaempferol-3-O-β-D-glucopyranoside with the retention time of 5.92 min, and its fragmentation pathways in negative ion mode was listed in Figure S2 (19, 20). Following the same pattern, compounds 1, 2, 3, 5, and 6 were identified as apigenin-7-O-β-D-glucoside, epicatechin, quercetin 3-O-β-D-glucuronide, scutellarin, and quercitrin with the retention time of 4.09, 4.55, 5.03, 6.63, and 9.62 min, respectively.

### PDBW Decreased Glucose Level in HFD/STZ-Induced Diabetic Mice

**Figure 2:**
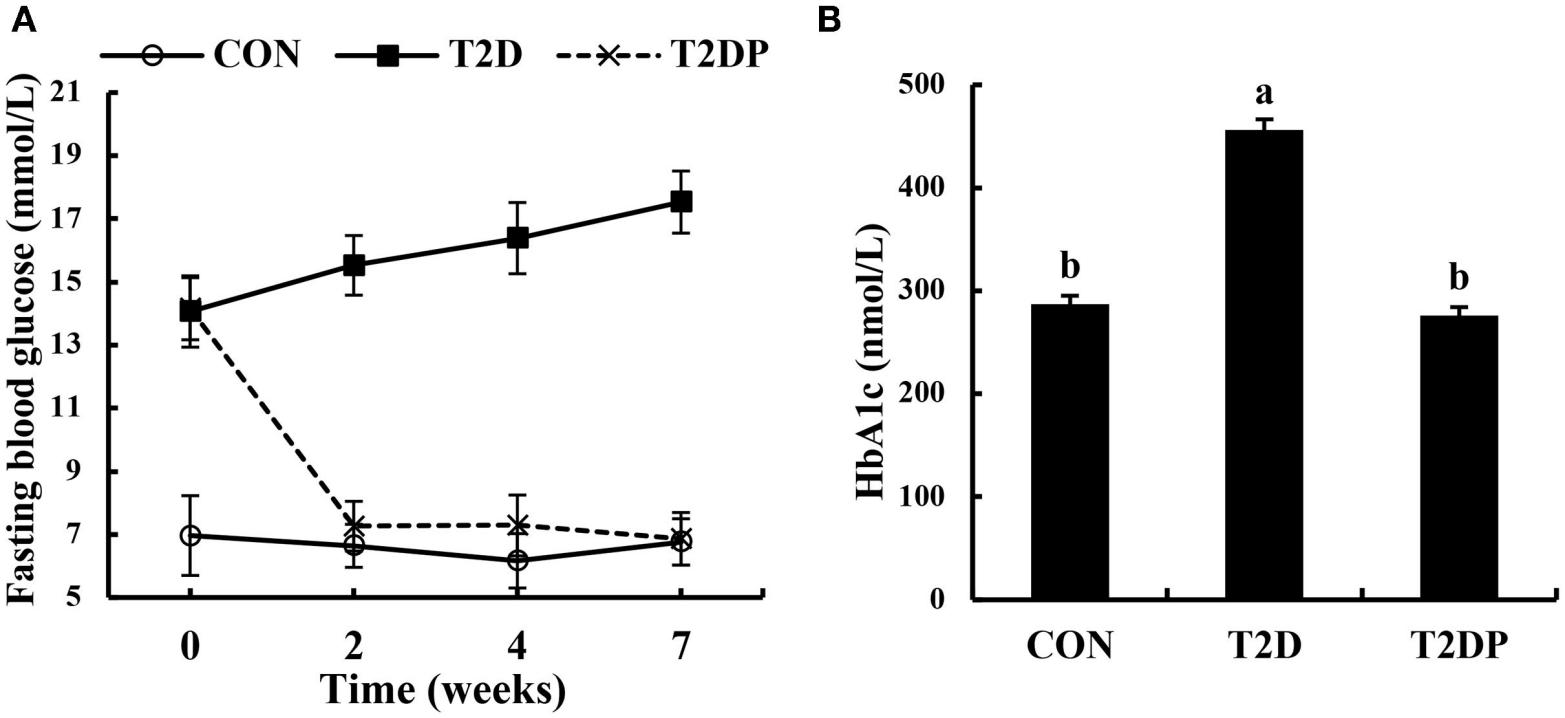
Effects of *Potentilla discolor* Bunge water extract (PDBW) on fasting blood glucose and HbA1c levels in HFD-STZ induced diabetic mice. **(A)** The levels of fasting blood glucose after PDBW treatment. **(B)** The levels of HbA1c after 8 weeks treatment with PDBW. All values are means ± SEM (*n* = 8); Values marked with different lower-case letters in superscript format indicate significant differences among groups (*p* < 0.05).

The blood glucose levels were measured after PDBW oral administration for 2, 4, and 7 weeks. As shown in Figure 2A, diabetic mice showed a higher blood glucose level compared to that in the CON group (*p* < 0.05). After oral administration of PDBW, blood glucose level was significantly reduced in comparison with that of T2D group (*p* < 0.05, Figure 2A). In line with the blood glucose level, T2D group had higher HbA1c level compared to the CON group while PDBW administration significantly decreased the HbA1c level (*p* < 0.05, Figure 2B).

### PDBW Ameliorated Body Weight Loss and Dyslipidemia in HFD/STZ-Induced Diabetic Mice

As shown in Table 1, compared to the CON group, T2D group showed lower body weight, while PDBW treatment to T2D mice increased body weight (*p* < 0.05). Meanwhile, there was no significant difference in the amount of food intake among the three groups (Table 1). The T2D group presented a lower insulin level than that of the CON group. Compared to T2D group, PDBW improved the insulin level by 53.3% (*p* < 0.05). T2D mice exhibited a higher level of TG, TC, LDL-c, and FFA and a lower level of HDL-c in serum than that of the CON group (*p* < 0.05), while PDBW treatment reversed these effects with a lower levels of TG, TC, LDL-c and FFA, and a higher level of HDL-c level in T2D mice (*p* < 0.05). Moreover, compared to the CON group, T2D group showed a higher level of AST and ALT in serum, whereas PDBW treatment improved the level of ALT and AST in T2D mice.

**Table 1:** Effects of *Potentilla discolor* Bunge water extract (PDBW) on body weight, food intake, and serum biochemical profiles.

	**CON**	**T2D**	**T2DP**
Initial body weight (g)	24.80 ± 0.58^a^	24.93 ± 0.31^a^	24.59 ± 0.38^a^
Body weight before PDBW treatment (g)	30.76 ± 0.31^a^	28.20 ± 0.42^b^	28.85 ± 0.41^b^
Final body weight (g)	32.82 ± 0.53^a^	28.77 ± 0.35^b^	31.33 ± 0.68^a^
Food intake (g/d)	3.75 ± 0.15^a^	3.79 ± 0.15^a^	3.74 ± 0.17^a^
Insulin (μg/L)	0.48 ± 0.05^a^	0.30 ± 0.034^b^	0.46 ± 0.07^a^
TG (mmol/L)	0.85 ± 0.11^c^	2.85 ± 0.06^a^	1.86 ± 0.07^b^
TC (mmol/L)	2.72 ± 0.19^c^	4.60 ± 0.10^a^	3.77 ± 0.1^b^
HDL-c (mmol/L)	1.68 ± 0.10^a^	0.93 ± 0.04^c^	1.09 ± 0.04^b^
LDL-c (mmol/L)	0.97 ± 0.05^c^	1.89 ± 0.08^a^	1.32 ± 0.05^b^
FFA (mmol/L)	0.90 ± 0.09^c^	3.80 ± 0.11^a^	2.75 ± 0.06^b^
AST (nmol/min/mL)	110.42 ± 13.53^b^	180.46 ± 20.42^a^	140.66 ± 15.32^a^
ALT (nmol/min/mL)	70.34 ± 6.75^c^	150.67 ± 19.46^a^	102.34 ± 14.23^b^

*All values are the mean ± standard error (n = 8); Values marked with different lower-case letters in superscript format indicate significant differences between three groups (p < 0.05)*.

### PDBW Improved Glucose Tolerance and Insulin Sensitivity in HFD/STZ-Induced Diabetic Mice

**Figure 3:**
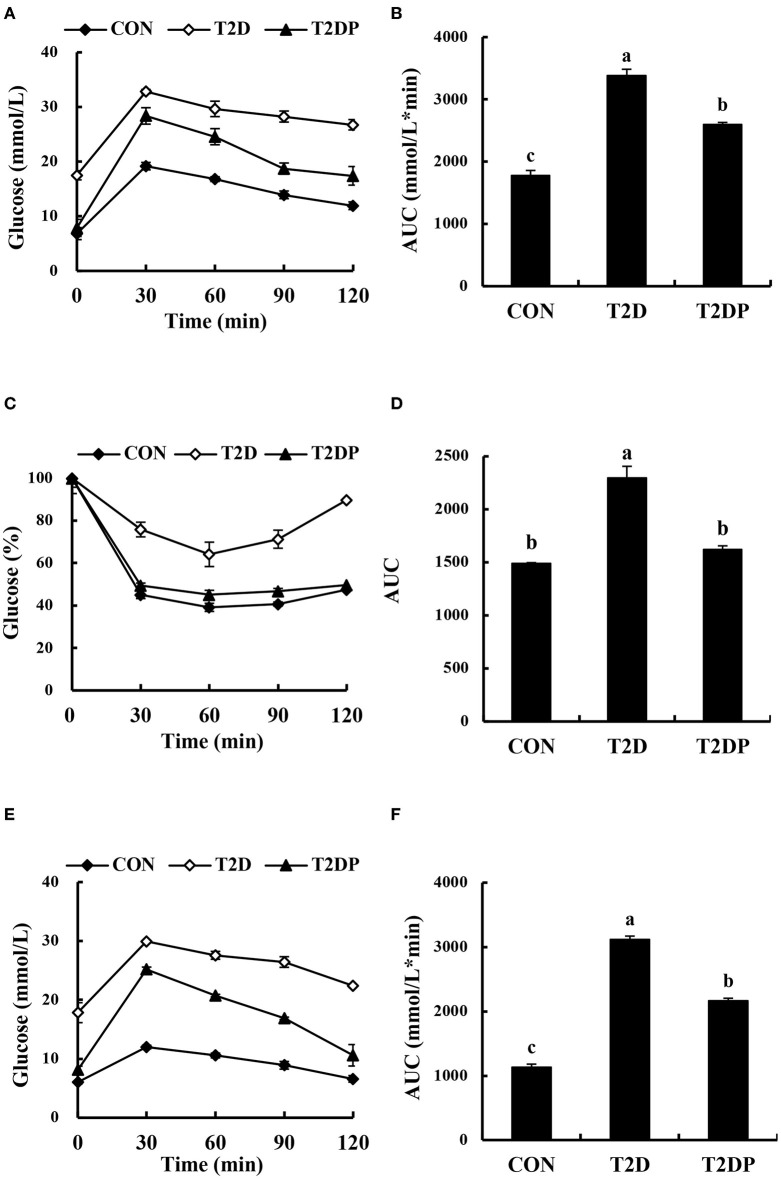
Effects of *Potentilla discolor* Bunge water extract (PDBW) on oral glucose tolerance (OGTT), intraperitoneal insulin tolerance (IPITT), and pyruvate tolerance test (PTT) in HFD-STZ induced diabetic mice. Blood glucose levels **(A)** and area under the curve (AUC) **(B)** for the blood glucose levels during OGTT. Blood glucose levels **(C)** and AUC **(D)** for the blood glucose levels during IPITT. The graph displays blood glucose levels expressed as a percentage of the initial blood glucose level following an overnight fast. Blood glucose levels **(E)** and AUC **(F)** for the blood glucose levels during PTT. All values are the mean ± standard error (*n* = 8); Values marked with different lower-case letters in superscript format indicate significant differences between three groups (*p* < 0.05).

To investigate the effect of PDBW on glucose tolerance, insulin tolerance and pyruvate tolerance, the OGTT, IPITT, and PTT were carried out. As shown in Figures 3A,B, fasting blood glucose was significantly increased in the T2D mice compared to that of the CON group mice (*p* < 0.05). The T2DP group, by contrast, showed a decrease in fasting blood glucose levels compared to the T2D group (*p* < 0.05). The blood glucose levels of all groups showed an increase and reached the highest at 30 min after glucose oral administration, and then decreased until the end. The area-under-the-curve (AUC) of T2D group during OGTT was significantly higher than that of the CON group (*p* < 0.05). PDBW treatment reduced the AUC compared to T2D group (*p* < 0.05), suggesting a reverse in impaired glucose tolerance induced by HFD/STZ. The curves of blood glucose vs. time in the IPITT showed that the blood glucose levels of mice from all groups bottomed at 60 min after insulin injection and then increased until the end (Figures 3C,D). The blood glucose levels of the mice in T2DP group were lower than those of T2D group at all the time points in IPITT. The AUC of T2D group was significantly higher than that of the CON group (*p* < 0.05) while PDBW treatment significantly decreased the AUC (*p* < 0.05) and improved insulin tolerance.

The effect of PDBW on PTT was presented in Figures 3E,F. The pyruvate tolerance capacity of the T2D group was severely impaired comparing with the CON group, as shown by the increase in blood glucose levels and higher AUC of PTT after sodium pyruvate solution injection. When the T2D mice were supplemented with PDBW, the blood glucose level and the AUC during PTT were markedly reduced (*p* < 0.05), suggesting the improvement in gluconeogenesis.

### PDBW Prevented Hepatic Lipid Accumulation in HFD/STZ-Induced Diabetic Mice

**Figure 4:**
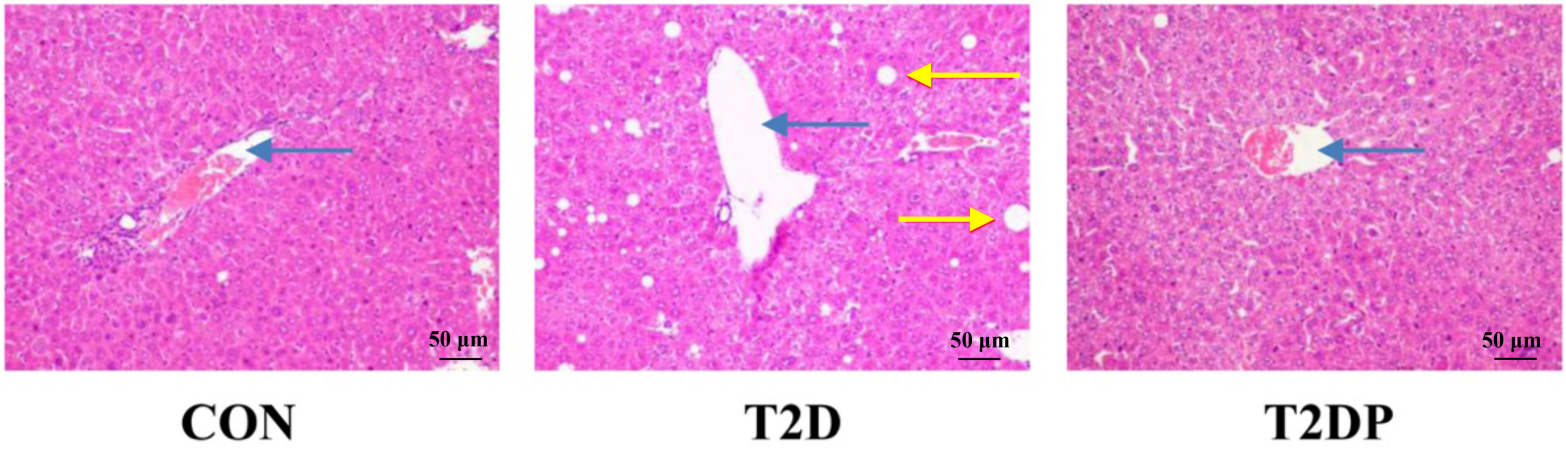
Effects of *Potentilla discolor* Bunge water extract (PDBW) on histological changes in liver (H&E, 200×) of HFD-STZ induced diabetic mice. Blue arrows indicate central veins. Yellow arrows indicate lipid droplets.

To investigate the effects of PDBW on the lipid accumulation of liver in HFD-STZ induced diabetic mice, H&E staining of liver tissues was performed. Histologic analysis of liver showed a remarkable increase in the amount of lipid vacuoles within hepatocytes in mice from the T2D group compared to the CON group (Figure 4). However, treatment of PDBW significantly decreased lipid droplets, suggesting PDBW effectively prevents hepatic lipid accumulation.

### PDBW Down-Regulated mRNA Expressions of Key Gluconeogenic Enzymes in Liver of HFD/STZ-Induced Diabetic Mice

**Figure 5:**
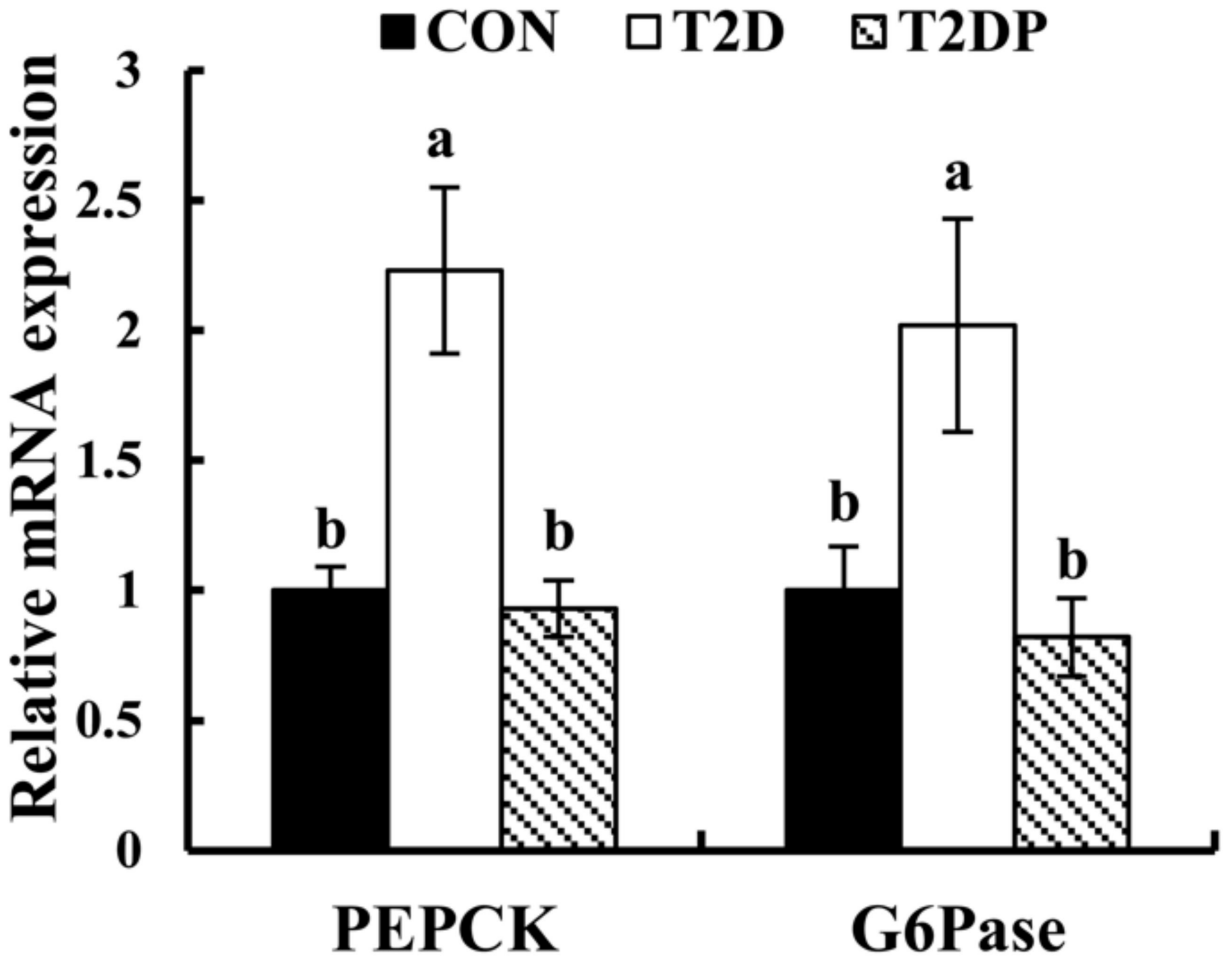
Effects of *Potentilla discolor* Bunge water extract (PDBW) on the phosphoenolpyruvate carboxykinase and glucose-6-phosphatase mRNA gene expression in liver of HFD-STZ induced diabetic mice. All values are the mean ± standard error (*n* = 6); Values marked with different lower-case letters in superscript format indicate significant differences between three groups (*p* < 0.05).

To investigate the effects of PDBW on gluconeogenesis in HFD-STZ induced diabetic mice, the mRNA expression of key gluconeogenic enzymes (PEPCK and G6Pase) was determined. As revealed in Figure 5, compared to the CON group, the mRNA expression levels of PEPCK and G6Pase were significantly increased in T2D group (*p* < 0.05). After PDBW treatment, the mRNA expression levels of PEPCK and G6Pase were decreased by 63.13 and 68.03%, respectively, compared to the T2D group (*p* < 0.05). The data suggests that PDBW might modulate the glucose gluconeogenesis through down-regulation of key enzymes involved in liver gluconeogenesis.

### PDBW Regulated Hepatic Glycogen Content and Phosphorylation Levels of Key Glycogenic Enzmyes in Liver of HFD/STZ-Induced Diabetic Mice

**Figure 6:**
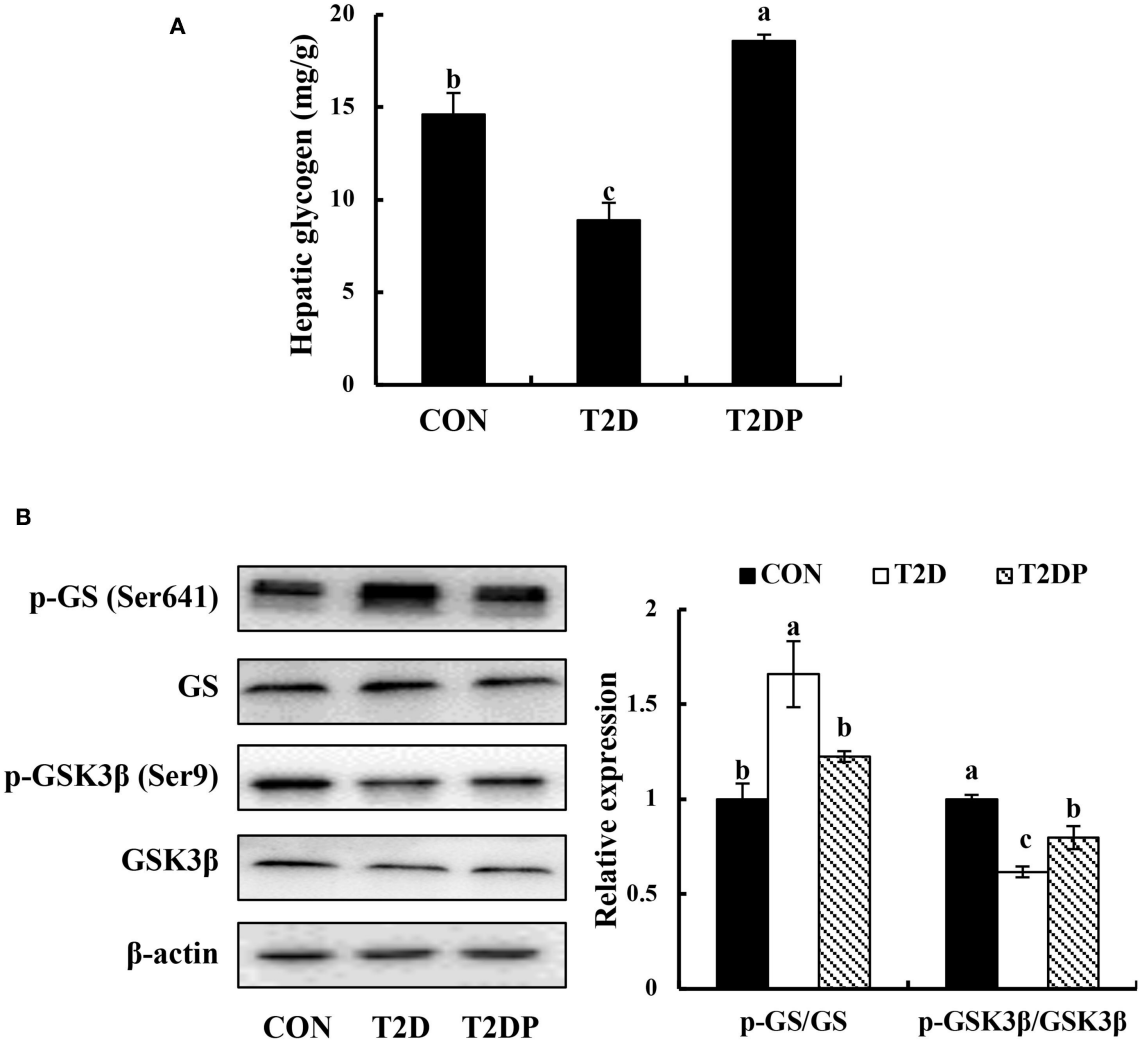
Effects of *Potentilla discolor* Bunge water extract (PDBW) on glycogenesis in liver of HFD-STZ induced diabetic mice. **(A)** Hepatic glycogen content. **(B)** Hepatic protein expression of p-GSK3β, GSK3β, p-GS, and GSK3β. All values are the mean ± standard error (*n* = 6); Values marked with different lower-case letters in superscript format indicate significant differences between three groups (*p* < 0.05).

To investigate the effects of PDBW on glycogenesis, the glycogen content and protein expression of enzymes involved in glycogen synthesis were determined. As shown in Figure 6A, compared to the CON group, the glycogen content was significantly reduced in diabetic mice of T2D group. However, PDBW supplementation increased the glycogen content by 108.64% compared to T2D group. As shown in Figure 6B, the ratio of p-GS/GS in T2D group was remarkably higher than that in the CON group (*p* < 0.05). After treatment with PDBW, the p-GS/GS ratio was significantly decreased (*p* < 0.05), suggesting the inhibition of GS phosphorylation. Besides, diabetic mice showed a lower p- GSK3β/GSK3β ratio in comparison with the CON group (*p* < 0.05). PDBW elevated the ratio of p-GSK3β/GSK3β, showing the increased phosphorylation of GSK3β. These results indicate that PDBW increased glycogen content by regulating the phosphorylation of GS and GSK3β in diabetic mice.

### PDBW Activated the PI3K/Akt and AMPK Signaling in Liver of HFD/STZ-Induced Diabetic Mice

**Figure 7:**
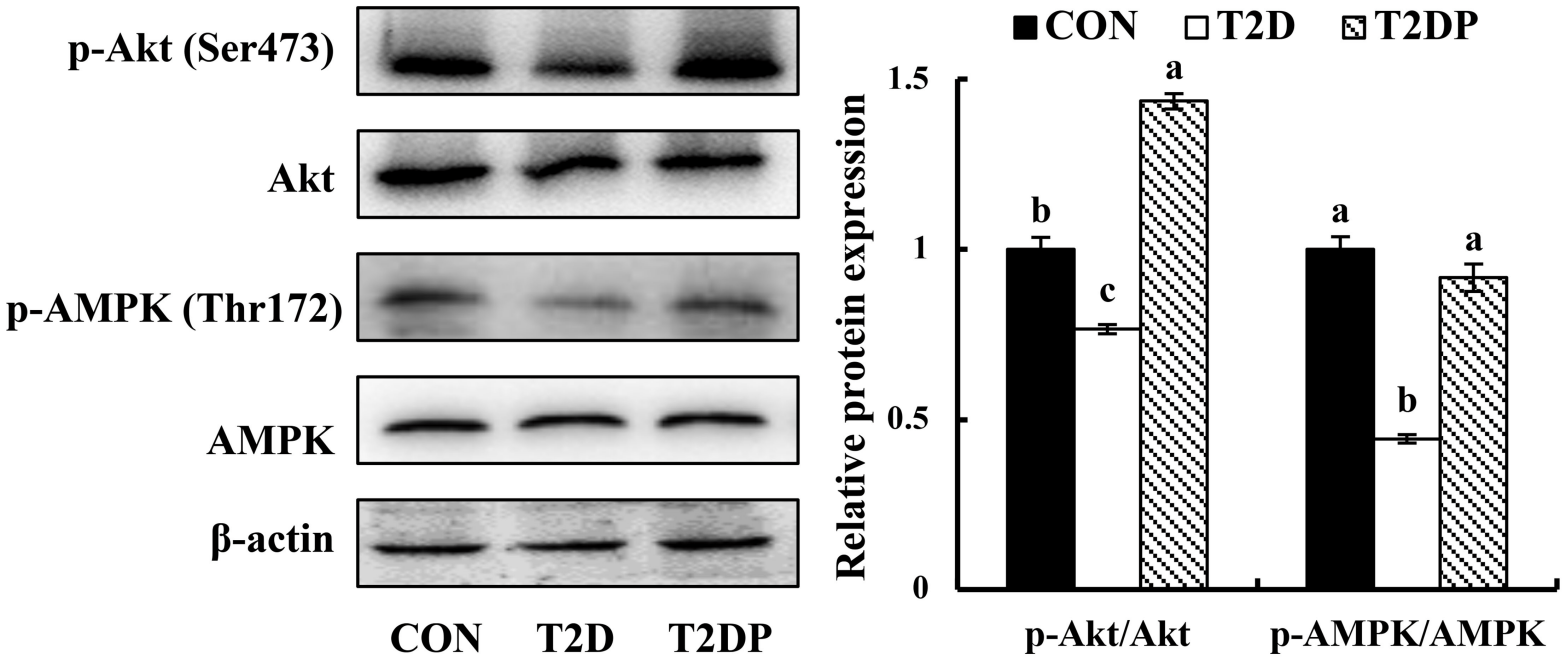
Effects of *Potentilla discolor* Bunge water extract (PDBW) on hepatic protein contents of p-Akt, Akt, p-AMPK, and AMPK. All values are the mean ± standard error (*n* = 6); Values marked with different lower-case letters in superscript format indicate significant differences between three groups (*p* < 0.05).

To further explore whether PDBW regulates gluconeogenesis and glycogenesis by activating Akt and AMPK pathway, the contents of p-Akt and p-AMPK were assessed. As shown in Figure 7, compared to the CON group, the ratio of p-Akt/Akt and p-AMPK/AMPK were reduced in T2D group (*p* < 0.05), while PDBW treatment increased the ratio of p-Akt/Akt and p-AMPK/AMPK. This result suggests that the effects of PDBW on the gluconeogenesis and glycogenesis are related to the activation of Akt and AMPK pathways.

## Discussion

Type 2 diabetes, one of the most common metabolic disorders, is associated with an abnormal modulation of glucose metabolism. Therefore, effective blood glucose control improves the living quality of T2D patients. In the present study, we found that PDBW decreased blood glucose level and increased serum insulin level, and improved glucose tolerance, insulin sensitivity and lipid profiles in HFD/STZ-induced diabetic mice. PDBW also regulated the liver glucose metabolism by inhibiting gluconeogenesis and increasing glycogen synthesis, which contributed to the alleviation of metabolic disorders in T2D.

In T2D, hyperglycemia is the consequence of insufficiency of insulin secretion from the pancreatic β cells and inability of target organs to respond to insulin (21). Afterwards, pancreas β cells succumb to the consistent high glucose level, leading to a series of metabolic syndrome (22). In order to initiate the insulin dysregulation related to T2D, the high-fat diet to animals is an effective method to induce obesity, which acts as a known risk factor for T2D (23). Besides, a low dose of β cell toxin STZ can cause a mild impairment of insulin secretion attributed to the later stage of T2DM and is often used to hasten the T2D development in mice similar to the condition observed in human (23). Therefore, a murine model of T2D induced by high fat diet combined with multiple low doses of STZ could mimic the metabolic characteristics of type 2 diabetes in humans (24). In our study, the HFD/STZ-induced diabetic mice exhibited high blood glucose concentrations and low plasma insulin levels with impaired glucose tolerance and insulin sensitivity, which are in concert with previous studies (25).

After PDBW treatment, the HFD/STZ-induced diabetic mice showed a significant decrease in blood glucose level and an increase in serum insulin level, paralleling the increase in insulin sensitivity shown by greater improvement in OGTT and IPITT. Another report also showed that treatment of ob-db mice with PDB decoction for 4 weeks caused a decrease in the blood glucose values (16). Besides, HbA1c reflects long-term glycaemic exposure and has better pre-analytical stability for diabetes than single measures of glucose concentration such as fasting blood glucose or OGTT (26). In our study, HbA1c of diabetic mice was remarkably increased while PDBW prevented the elevation, indicating the anti-diabetic effects of PDBW. It has been reported that some of the major compounds of PBDW could attenuate hyperglycemia and related metabolic disorders. Epicatechin has been shown to lower blood glucose levels in diabetic patients and restored insulin sensitivity and improved glucose metabolism in HFD-fed mice (27). Cyclocarya paliurus with a high content of quercetin 3-O-β-D-glucuronide was beneficial to reverse body weight loss and to reduce glucose levels in OGTT and IPITT tests of STZ-induced diabetic mice (28). Pilea microphylla rich in apigenin-7-O-β-D-glucoside produced significant reduction in plasma glucose in HFD/STZ-induced diabetic mice (29). Besides, α-glucosidase inhibitors are used and marketed as anti-diabetic drugs that can prevent carbohydrates from producing glucose (30). Quercetin 3-O-β-D-glucuronide and apigenin-7-O-glucoside exhibited remarkable α-glucosidase inhibitory activity and might serve as effective α-glucosidase inhibitor and insulin sensitizer (31). Therefore, the anti-hyperglycemic effects of PDBW may involve in these bioactive compounds-induced improvement in blood glucose control.

Notably, hyperlipidemia is closely related with hyperglycemia in diabetic patients with poor glucose metabolic control and increase the risk of diabetic vascular complications (5). Previous studies have demonstrated that HFD-fed mice become more sensitive to the development of hyperlipidemia under STZ treatment (25). In the present study, diabetic mice had a significant increase in TG, TC, LDL-c, and FFA levels and a decrease in HDL-c level while PDBW administration reversed these effects. Additionally, the H&E staining of liver showed that PDBW treatment significantly reduced lipid accumulation, demonstrating that PDBW prevented the abnormalities in lipid metabolism. Consistent with our results, the major component of PDBW, epicatechin, alleviated liver fat accumulation and reduced the contents of TC, LDL-c, and TG while increased HDL-c in hyperlipidemic rats (32). Scutellarin given to patients with hyperlipidemia decreased the levels of TC, LDL-c, and TG, but increased the level of HDL-c (33). The flavonoid rich fraction containing apigenin-7-O-β-D-glucoside decreased the TG and TC contents in plasma of HFD/STZ-induced diabetic mice (29).

The liver is primarily responsible for the maintenance of blood glucose levels by its ability to produce glucose from gluconeogenesis and to store glucose as glycogen (6). Hepatic glucose output is primarily regulated by PEPCK and G6Pase, which are the rate-limiting enzymes in gluconeogenesis (34). In the present study, PDBW reduced the elevated mRNA levels of PEPCK and G6Pase induced by HFD/STZ. Moreover, PDBW-treated diabetic mice exhibited significant decrease in blood glucose levels after an injection of pyruvate, indicating that gluconeogenesis from pyruvate decreased *in vivo*. Glycogen synthase is a key rate-limiting enzyme for glycogen synthesis, which catalyzes the incorporation of UDP-glucose into glycogen chains. GSK3β has been implicated in mediating the development of insulin resistance, mainly by inhibition of glycogen synthesis (35). GSK3β inhibits glycogen biosynthesis through inactivation of GS by inhibitory phosphorylation (35). In our study, PDBW improved accumulation of hepatic glycogen in T2D mice, as evidenced by the raise in the hepatic glycogen contents. PDBW treatment reversed the elevation in the phosphorylation level of GS and up-regulated the phosphorylation level of GSK3β, indicating that PDBW promoted glycogenesis. These findings are similar to previous reports showing that epicatechin possessed insulin-like effects and modulated the expression of PEPCK, leading to a diminished hepatic glucose production (36). Quercitrin increased the insulin secretion and improved glucose homeostasis along with the restoration of glycogen content and alteration of gluconeogenic enzymes in streptozotocin-induced diabetic rats (37).

In relation to T2D, many protein kinases have been shown to play vital roles in the regulation of hepatic glucose metabolism and insulin sensitivity, such as Akt and AMPK. The serine/threonine kinase Akt, also known as protein kinase B (PKB), plays a central but diverse role in cell signaling downstream of hormones, growth factors, cytokines, and other cellular stimuli (10). Insulin-mediated regulation of glycogenesis and gluconeogenesis are associated with the phosphorylation of insulin receptor substrate mediated by the insulin receptor, thus activating the PI3K/Akt signal pathway (38). The impairment of signaling through PI3K/Akt may predispose to the development of diabetes (38). Besides, AMPK, the key regulator of energy balance, has the potential to control whole-body glucose metabolism against obesity and T2D (12). Particularly, the activation of AMPK can lead to a decrease in gluconeogenesis-related gene transcription, increased fatty acid oxidation, and decreased fatty acid synthesis in liver, thereby lowering the blood glucose level and lipid accumulation (39). Epicatechin from cocoa strengthened the insulin signaling by Akt phosphorylation and suppressed hepatic gluconeogenesis through AMPK activation in HepG2 cells (36). Quercetin 3-O-β-D-glucuronide ameliorated insulin resistant endothelial dysfunction by positive regulation of Akt (40). Scutellarin could promote glucose uptake in adipocytes by management of AMPK or Akt activity (41). Herein PDBW increased the phosphorylation of both Akt and AMPK in HFD/STZ mice, which demonstrated that PDBW provided strong impacts on insulin signaling regulation by Akt and AMPK activation.

Many metabolomics studies have showed that the metabolites including glucose, pyruvate, lactate, β-hydroxybutyrate, succinate, citrate, and 2-oxoglutarate have been generally identified as biomarkers in diabetic models and most of them are related to the carbohydrate metabolism, particularly glycolysis/gluconeogenesis and TCA cycle (42). Glucose can be broken down and converted into pyruvate that is closely correlated with the glycolysis/gluconeogenesis pathway (42). The increased level of glucose suppresses the action of glycolytic enzymes and activates gluconeogenesis metabolism, thus reducing the pyruvate levels in the diabetic animal models (43, 44). Then the decrease in pyruvate level reduces the acetyl-CoA production and results in the reduction of TCA cycle intermediates (45). Administration of several medicinal plants can prevent metabolic disorders in diabetic rats, which was always associated with the alternations of metabolic intermediates linked to glucose metabolism and TCA cycle (46, 47). Therefore, PBDW may contribute to the antidiabetic activity through regulating the metabolites associated with glycolysis/gluconeogenesis and TCA cycle. However, the specific metabolites linked to glucose metabolism altered by PDBW need to be further investigated by metabolomics.

## Conclusion

To conclude, oral PDBW administration at 400 mg/kg BW prevented the decrease of body weight, reduced the levels of blood glucose and HbA1c and increased serum insulin levels in HFD-STZ induced diabetic mice. The lipid profiles, glucose tolerance and insulin sensitivity were improved after PDBW treatment. PDBW regulated gluconeogenesis by decreased the mRNA expression of PEPCK and G6Pase. PDBW also promoted glycogenesis as shown by the increase in hepatic glycogen content and GS phosphorylation and the down-regulation of GSK3β phosphorylation. Furthermore, the upstream signaling pathways, Akt and AMPK, may mediate the effects of PDBW on hepatic glucose metabolism. These findings provide evidences of PDBW in the prevention and amelioration of type 2 diabetes.

## Data Availability Statement

The raw data supporting the conclusions of this article will be made available by the authors, without undue reservation.

## Ethics Statement

The animal study was reviewed and approved by China Agricultural University Animal Ethics Committee.

## Author Contributions

TL, RC, and XM contributed to the conception and design of the study. TL and RC conducted experiments. TL and HZ analyzed the data. TL wrote the manuscript. XM and MD revised the manuscript. All authors contributed to the article and approved the submitted version.

## Conflict of Interest

The authors declare that the research was conducted in the absence of any commercial or financial relationships that could be construed as a potential conflict of interest.
