# Supplementary material for: Water Extract of Potentilla discolor Bunge Improves Hepatic Glucose Homeostasis by Regulating Gluconeogenesis and Glycogen Synthesis in High-Fat Diet and Streptozotocin-Induced Type 2 Diabetic Mice
Source: Front Nutr. 2020 Sep 15;7:161. doi: 10.3389/fnut.2020.00161 (PMC7522508; doi:10.3389/fnut.2020.00161)
Supplement: Supplementary file 1 [file Data_Sheet_1.docx]

Supplementary Material

# Supplementary Figures and Tables

## Supplementary Figures

**
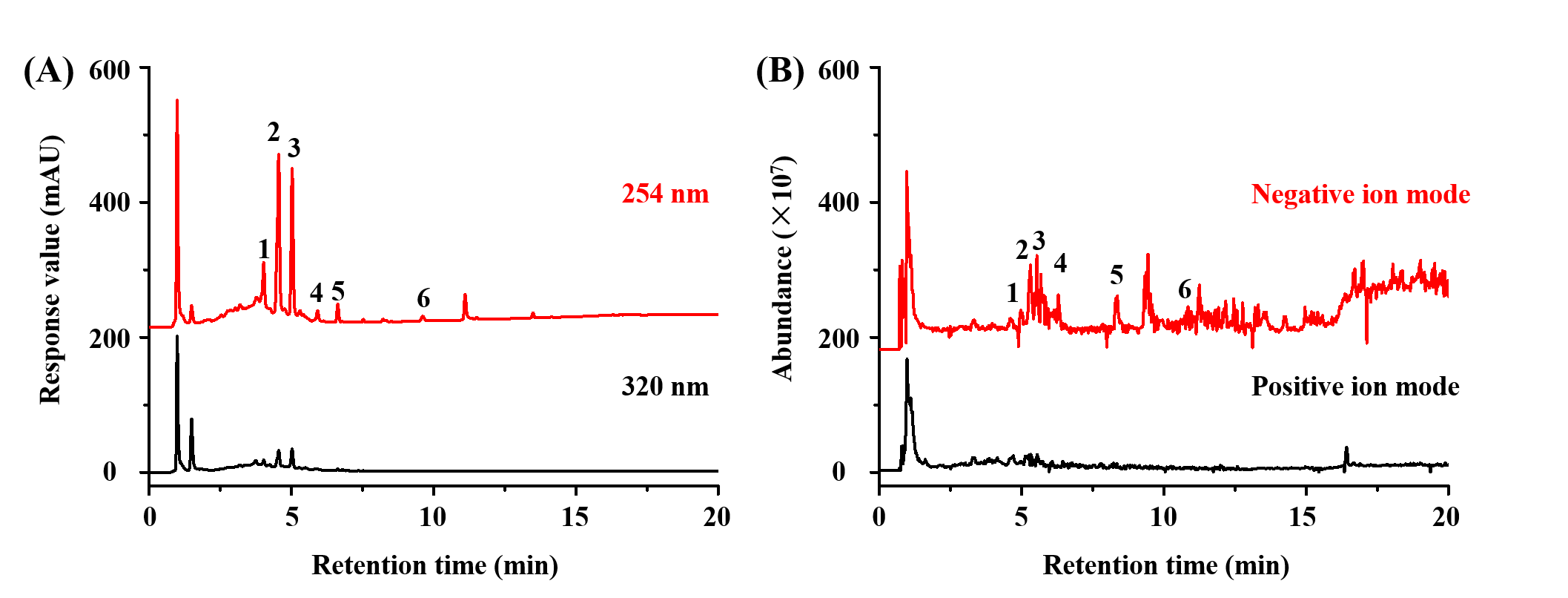
**

**Supplementary Figure 1.** Composition analysis of the PBD aqueous extract sample. (A) LC profiles under the UV wavelength of 254 nm (in red) and 320 nm (in black), (B) the MS total ion chromatogram in the positive (in red) and negative (in black) ion mode.


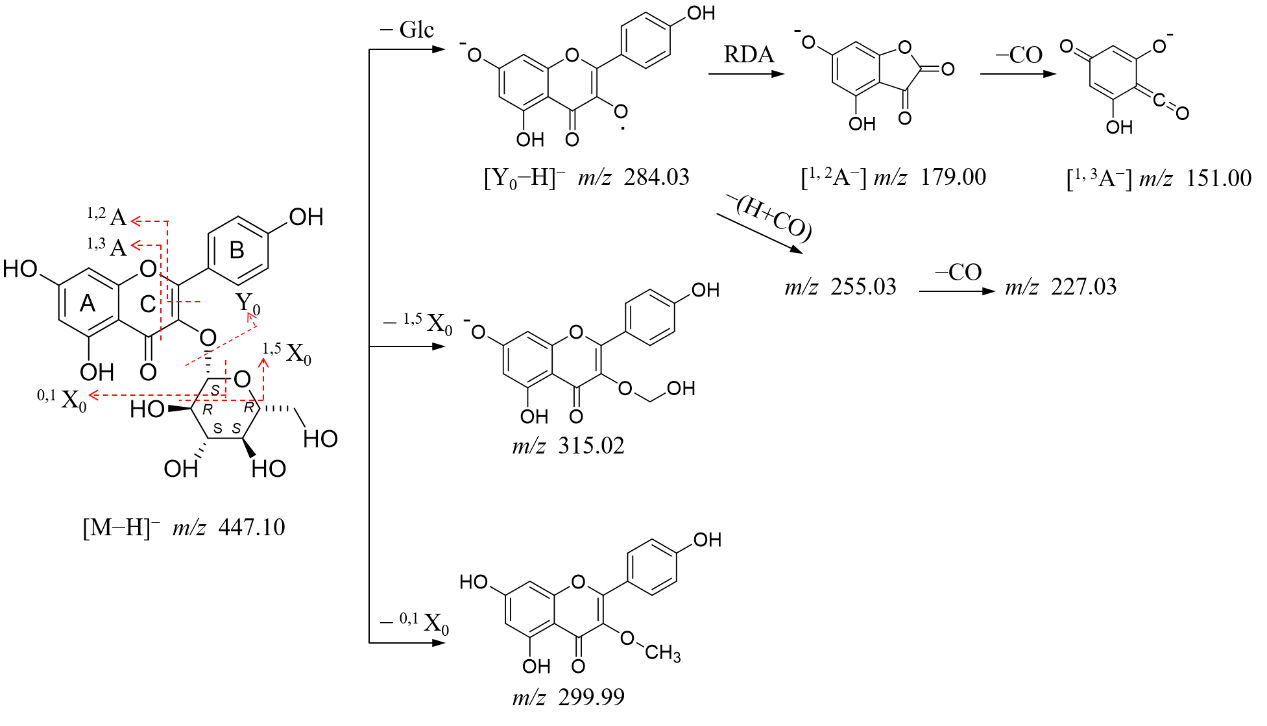


**Supplementary Figure 2.** Fragmentation pathways of Kaempferol-3-O-β-D-glucopyranoside (compound 4) in negative ion ESI-MS/MS.

## Supplementary Table

**Supplementary Table 1. Primers used for RT-PCR**

| Gene | Forward Primer (5’-3’) | Reverse Primer (5’-3’) |
| --- | --- | --- |
| PEPCK | TCATCATCACCCAAGAGCA | CCACCACATAGGGCGAGT |
| G6Pase | ATCAATCTCCTCTGGGTGGC | TGTTGCTGTAGTAGTCGGTGTCC |
| GAPDH | TGGCCTCCAAGGAGTAAGAAAC | TGGCCTCCAAGGAGTAAGAAAC |
